# Altered Resting-State Functional Connectivity of Striatal-Thalamic Circuit in Bipolar Disorder

**DOI:** 10.1371/journal.pone.0096422

**Published:** 2014-05-02

**Authors:** Shin Teng, Chia-Feng Lu, Po-Shan Wang, Cheng-Ta Li, Pei-Chi Tu, Chih-I Hung, Tung-Ping Su, Yu-Te Wu

**Affiliations:** 1 Department of Biomedical Imaging and Radiological Sciences, National Yang-Ming University, Taipei, Taiwan, ROC; 2 Department of Physical Therapy and Assistive Technology, National Yang-Ming University, Taipei, Taiwan, ROC; 3 Department of Education and Research, Taipei City Hospital, Taipei, Taiwan, ROC; 4 Brain Research Center, National Yang-Ming University, Taipei, Taiwan, ROC; 5 The Neurological Institute, Taipei Municipal Gan-Dau Hospital, Taipei, Taiwan, ROC; 6 School of Medicine, National Yang-Ming University, Taipei, Taiwan, ROC; 7 Department of Psychiatry, Taipei Veterans General Hospital, Taipei, Taiwan, ROC; 8 Department of Medical Education and Research, Taipei Veterans General Hospital, Taipei, Taiwan, ROC; 9 Division of Psychiatry, Faculty of Medicine, National Yang-Ming University, Taipei, Taiwan, ROC; 10 Institute of Brain Science, National Yang-Ming University, Taipei, Taiwan, ROC; 11 Institute of Biophotonics, National Yang-Ming University, Taipei, Taiwan, ROC; Laureate Institute for Brain Research and The University of Oklahoma, United States of America

## Abstract

Bipolar disorder is characterized by internally affective fluctuations. The abnormality of inherently mental state can be assessed using resting-state fMRI data without producing task-induced biases. In this study, we hypothesized that the resting-state connectivity related to the frontal, striatal, and thalamic regions, which were associated with mood regulations and cognitive functions, can be altered for bipolar disorder. We used the Pearson's correlation coefficients to estimate functional connectivity followed by the hierarchical modular analysis to categorize the resting-state functional regions of interest (ROIs). The selected functional connectivities associated with the striatal-thalamic circuit and default mode network (DMN) were compared between bipolar patients and healthy controls. Significantly decreased connectivity in the striatal-thalamic circuit and between the striatal regions and the middle and posterior cingulate cortex was observed in the bipolar patients. We also observed that the bipolar patients exhibited significantly increased connectivity between the thalamic regions and the parahippocampus. No significant changes of connectivity related to the frontal regions in the DMN were observed. The changed resting-state connectivity related to the striatal-thalamic circuit might be an inherent basis for the altered emotional and cognitive processing in the bipolar patients.

## Introduction

Bipolar disorder is a mood disorder exhibiting a prevalence of at least 1%, constituting a considerable health care burden [1], [2]. Internally affective disturbances of the mental state, including mania and depression, are the principal symptoms of bipolar patients. Patients in a manic state typically experience an increase in energy, racing thoughts, and a decreased need for sleep. In a depressive state, patients experience sadness, guilt, hopelessness, disturbances to sleep and appetite, and a loss of interest in usually enjoyable activities. Additionally, bipolar patients have been reported to exhibit deficits in several cognitive functions, such as sustained attention, executive function, verbal memory, and decision making [3]–[6].

Emotional and cognitive processing, which are impaired in bipolar patients, are mediated by the frontal-striatal-thalamic (FST) circuit [7]–[9]. The FST circuit involves in diverse functions, including mood regulation, reward processing, action selections, strategic planning, and working memory. The functions of the FST circuit rely on the various interactions among the regions in the circuit [8], [10], [11]. For example, the thalamus participates in the FST circuit for relaying striatal inputs to the frontal regions and providing feedback to the striatum [7], [8]. Additionally, the medial frontal regions of the FST circuit are connected with the posterior cingulate cortex and the temporal regions in the resting state and these regions resemble the default mode network (DMN) which is implicated in emotional and self-referential processing [12]. The interactions between the FST circuit and the DMN regions may also be involved in the pathophysiology of bipolar disorder [9]. Although previous neuroimaging studies have reported structural and functional abnormalities in the regions in the FST circuit of bipolar patients [13]–[17], the interaction changes within the FST circuit and between this circuit and other regions have been explored less thoroughly.

Interaction between brain regions can be assessed using functional connectivity analysis, which is a method for estimating correlations of brain activity between regions. Resting-state functional connectivity analysis has been widely used to study the abnormalities of psychiatric diseases [18]–[21]. Performing functional connectivity analysis on resting-state data can reflect the natural mental state without producing task-induced biases and can facilitate investigating neural plasticity after long disease durations [18], [19]. Previous resting-state functional connectivity studies on bipolar disorder have focused on anterior cingulate cortex connectivity [21] and the DMN [19], which are involved in emotional and self-referential processing. Because the functional connectivity regarding the FST circuit in previous studies has been limited by the within-circuit connectivity using emotion-relevant stimuli [22]–[24], the resting-state connectivity in the FST circuit and between this circuit and other regions have not been addressed.

In this study, we hypothesized that the resting-state connectivity analysis can provide a task-unbiased observation to reveal the abnormality of inherently mental state for bipolar disorder. Based on the substantial studies that have reported that the bipolar patients exhibit deficits in mood regulations and cognitive functions, the altered functional connectivity may be related to the FST regions. Considering the facts that 1) the FST circuit was previously defined by the activated areas when performing emotional or cognitive tasks [8], [9] and 2) the medial frontal regions of the FST circuit were primarily involved in the DMN during resting state [12], we anticipated that the resting-state connectivities related to the striatal-thalamic circuit and DMN may be altered in bipolar patients. To determine whether the striatal-thalamic circuit and DMN exist in our resting-state data, we used the hierarchical modular analysis to cluster the 90 resting-state functional regions of interest (ROIs) [25] into several modules according to the strength of interregional connectivity [26], [27]. We then selected the ROIs in the modules associated with the striatal-thalamic circuit and DMN as the seed ROIs and subsequently compared the functional connectivity related to these seed ROIs between bipolar patients and healthy controls.

## Materials and Methods

### 2.1. Participants

The Institutional Review Board of Taipei Veterans General Hospital approved this study. All participants provided written informed consent before participating in this study. We recruited 15 patients with bipolar I disorder (mean age: 42.6 years; 10 men), characterized by at least one manic episode, from Taipei Veterans General Hospital. Clinical psychiatrists confirmed the diagnoses of all patients, using the fourth edition of the *Diagnostic and Statistical Manual of Mental Disorders* (DSM-IV). All of the bipolar patients exhibited no comorbidities, including schizophrenia, obsessive-compulsive spectrum disorders, and post-traumatic stress disorder. Before acquiring functional magnetic resonance images (fMRI), we rated the depressive and manic symptoms of bipolar patients by using the Hamilton Depression Rating Scale (HAM-D) [28] and the Young Mania Rating Scale (YMRS) [29], respectively. We required the bipolar patients to discontinue their medication for 1 week before imaging. The drug regimen for the bipolar group before their participation in this study included mood stabilizers, atypical antipsychotics, and hypnotic medications. We also recruited 16 age- and gender-matched healthy controls (mean age: 43.4 years; 11 men) presenting no personal or family history of psychiatric illness. All participants were right-handed and presented with no history of neurological or physical disorders, alcohol or drug dependence, or electroconvulsive therapy. Table 1 lists the demographic and clinical details of all participants.

**Table 1:** Demographic and clinical details.

	Healthy controls	Bipolar I patients	p-value
Number of subjects	16	15	
Gender (male/female)	11/5	10/5	0.938^a^
Age (years)†	43.4±11.3	42.6±9.7	0.827^b^
HAMD†	1.1±1.4	7.5±7.5	0.003^b^
YMARS†	0.0±0.0	3.9±4.5	0.002^b^
Duration of illness (years) †	**—**	17.3±11.5	
Onset age (years) †	**—**	25.3±10.8	
Past manic episodes (times) †	**—**	4.3±2.4	
Past depressive episodes (times) †	**—**	3.0±1.4	
Drug regimen before imaging			
Mood stabilizers		14	
Antipsychotics		12	
Hypnotic medications		12	

HAMD: Hamilton Depression Rating Scale, 17 items; YMARS: Young Mania Rating Scale;

† Continuous variables are expressed as mean ± standard deviation (SD);

a Pearson Chi-Square test;

b Two-tailed two-sample *t*-test.

### 2.2. Imaging Data Acquisition and Preprocessing

We performed functional magnetic resonance imaging (fMRI) by using the Discovery MR750 3T system (GE Healthcare, USA) at Taipei Veterans General Hospital. We scanned each participant in a supine position. All of the participants were instructed to close their eyes, hold still, relax, and not fall asleep. After the resting scan, all of the participants reported that they could comply with the directions. No other functional tasks were administered to the participants. We acquired the resting-state fMRI of the entire brain in 43 axial slices with 200 volumes by using an echo planar imaging (EPI) sequence. The imaging parameters included a repetition time (TR) of 2500 ms, echo time (TE) of 30 ms, flip angle of 90°, field of view (FOV) of 222×222 mm, in-plane resolution of 3.47×3.47 mm, and slice thickness of 3.5 mm. We also obtained structural 3D T1-weighted images by using a rapid acquisition gradient echo for each participant (TR = 12.2 ms, TE = 5.2 ms, flip angle = 12°, FOV = 256×256×168 mm^3^, and matrix size  = 256×256×168).

We implemented the preprocessing of functional analysis by using statistical parametric mapping (SPM8; http://www.fil.ion.ucl.ac.uk/spm/). We discarded the first 4 volumes of resting fMRI to exclude the magnetization equilibration effects and adaptation of the participants to the resting state. We corrected the remaining 196 volumes for acquisition time delay among slices and realigned the volumes for head-motion correction (a 6-parameter affine transformation). We registered all of the corrected images to the corresponding anatomical T1-weighted images, which were spatially normalized to the T1 template image with a voxel size of 2×2×2 mm. Finally, we applied a 6-mm full width at half maximum (FWHM) Gaussian kernel to perform spatial smoothing on the normalized images.

### 2.3. Functional Connectivity Between Pairs of 90 Functional Regions of Interest

We parcellated the brain into 90 functionally defined ROIs, based on a template proposed by Shirer et al (2012) [25]. Each ROI exhibited a volume of at least 30 mm^3^. Using functionally defined ROIs is superior to using anatomically defined ROIs when measuring brain functional connectivity. Because the brain features a complex functional organization that is composed of small functional units, averaging time courses over functional parcellation is more reliable than averaging them over large anatomical parcellation, such as the Automated Anatomical Labeling template [25], [30]. The details and abbreviations of the 90 functional ROIs are provided in Table S1.

We obtained the regional time series in each ROI by averaging the fMRI time series over all voxels in the ROI. To remove the bias of averaging signal to the voxels with high signal intensity, the fMRI time series in each voxel was normalized by its mean signal value. We regressed out the influence of head motion and the confounding effects of cerebrospinal fluid, white matter, and the global mean to eliminate the effects of physiological noise, such as respiratory-related fluctuations [31]–[33]. Because low-frequency components are the major contributors of resting-state functional connectivity [34], each regional time series was temporally bandpass filtered (0.01–0.08 Hz) to reduce the effects of low-frequency drift and high-frequency physiological noise. We calculated the functional connectivity between the filtered time series of each pair of ROIs by using Pearson's correlation, resulting in a 90 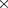 90 correlation matrix for each participant. We also applied Fisher's r-to-z transformation to ensure the normality of the correlation coefficients for subsequent statistical analysis [35].

### 2.4. Hierarchical Modular Analysis

To determine whether the striatal-thalamic circuit and DMN exist in our resting-state data, we applied hierarchical modular analysis to segregate the brain regions into several modules based on the 90 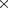 90 representative correlation map. The representative correlation map was derived from the following 3 steps: (1) the correlation matrices among all participants were firstly examined using a one-sample *t* test, (2) a Bonferroni-corrected significance level of *P*<0.001 was used to threshold the resulting *P* value matrix into a binary matrix for which the element was 1 if a significant correlation between a pair of ROIs existed and 0 otherwise, and (3) the values of 1 s in the binary matrix were replaced with the corresponding mean correlations among all correlation matrices.

In modular analysis, modules have the characteristics that the connections are stronger within each module and that they are weaker between modules [36]. The suitability of a modular partition can be measured by modularity, *Q* [37]:
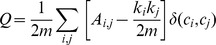
where *A_i,j_* is the correlation coefficient between regions *i* and *j*, 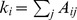 is the sum of the correlation coefficient between region *i* and its connected regions, *c_i_* is the module to which region *i* is assigned, the 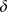-function 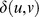 is 1 if *u* = *v* and 0 otherwise (i.e., when regions *i* and *j* belong to the same module, the 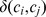 = 1), and 
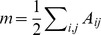
. Modularity *Q*, which ranges between −1 and 1, measures the density of links within modules compared with links between modules [36], [37]. A larger *Q* implies a superior partition that is more likely to constitute a modular organization [38].

Hierarchical modular analysis is an algorithm that facilitates modularity optimization [26], [27]. At the beginning of the algorithm, each region is assigned a different module. Two phases are then repeated. The first phase is a greedy optimization in which each region is designated to the same module or to an adjacent module. The assigned module for a particular region changes only if this action causes an increase in modularity. In the second phase, a new novel network comprising nodes that are the modules obtained from the first phase is constructed. A combination of the 2 phases is repeated until no further improvement of modularity is possible. When the maximal modularity is obtained, the optimal modular organization is determined.

Based on the results of the hierarchical modular analysis, we selected ROIs in the modules related to the frontal, striatal, and thalamic regions as the seed ROIs to investigate the alterations of resting-state functional connectivity in bipolar patients.

### 2.5. Statistical Analysis for Group Comparison

To identify whether functional connectivity is significantly different from zero in each group, we applied a one-sample *t* test (*P*<0.001 with false discovery rate (FDR) correction for multiple comparisons [39]) to each functional connectivity among participants in the bipolar and healthy groups. For example, once 11 seed ROIs related to the striatal-thalamic circuit were identified (see Section 3.1 for the results of hierarchical modular analysis), each element of an 11 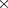 90 connectivity matrix (including the inter-seed-ROIs connectivity, represented by an 11 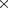 11 matrix, and the connectivity between seed ROIs and other ROIs, represented by an 11 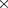 79 matrix) was tested by one-sample *t* test to statistically confirm the existence of functional connectivity for each group. After performing the one-sample *t* test, we obtained the functional connectivity exhibiting strong correlations (positive or negative; i.e., nonzero) that were consistent among participants in the bipolar and healthy groups. We subsequently examined the significant difference of the nonzero functional connectivity between the bipolar and healthy groups using the two-sample *t* test as *P*<0.05 with an FDR correction for multiple comparisons [39].

### 2.6. Clinical Correlates Analysis

We further examined the relationship between the *n* significantly altered the functional connectivity of bipolar patients and 3 clinical rating scales (i.e., the HAM-D, YMRS scores, and the duration of illness) by using a partial correlation controlling for age and gender effects [40]. The FDR correction for multiple comparisons (*n* 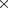 3) was performed on the correlation between the altered connectivity and clinical rating scales. The significant correlation (*P*<0.05; FDR correction) between each clinical rating scale and functional connectivity indicated that the specific connectivity strength might reflect the degree of depression (HAM-D score) or mania (YMRS score), and duration of illness in bipolar patients.

## Results

### 3.1. Regions in the Striatal-thalamic Circuit Were Clustered as a Module

**Figure 1:**
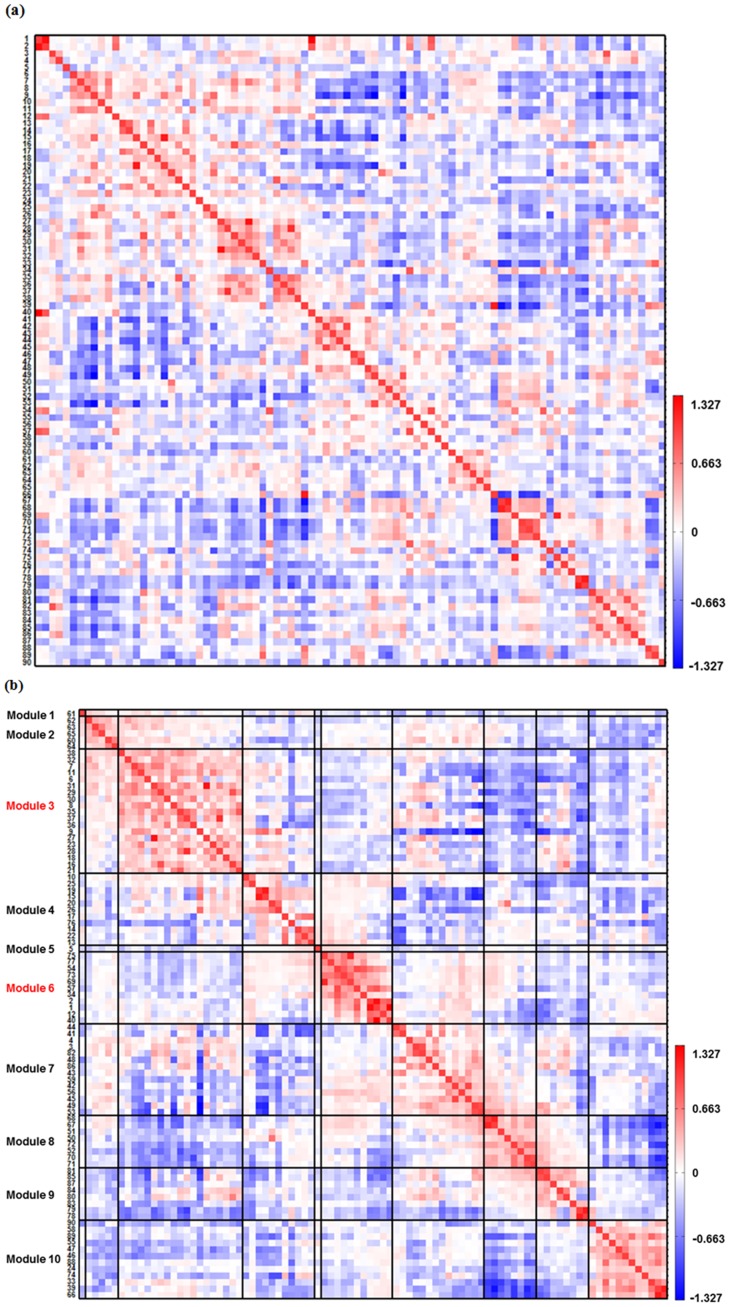
The mean functional connectivity across all participants. (a) The mean 90 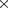 90 correlation matrix across all participants. The anatomical locations of 90 ROIs are listed in the Table S1. The warm colors represent the positive correlations whereas the cool colors represent the negative correlations between ROIs. (b) The modular structure of brain functional connectivity across all participants. This modular pattern is obtained by reordering regions in the mean correlation matrix according to maximizing the strength of connectivity close to the main diagonal of the matrix. Module 6 consisted of 3 striatal regions and 8 thalamus subregions forming the striatal-thalamic circuit. Module 3 consisted of 9 medial frontal regions, 1 temporal region, 7 parietal regions, 1 posterior cingulate cortex and 1 occipital region forming the DMN. The details of the modular structure are listed in Table S2.

Based on the results of the hierarchical modular analysis, the 90 ROIs were segregated into 10 modules. We reordered the regions in the mean correlation matrix among all of the participants (Figure 1a) to illustrate the modular structure in which the strength of connectivity near the diagonal of the matrix was strong (Figure 1b). Eleven ROIs, including 3 striatal regions (the ventral and dorsal caudate and dorsal putamen) and 8 thalamic subregions (the anterior, mediodorsal, ventral posterolateral, and pulvinar thalamus) were clustered in one module, namely, the striatal-thalamic circuit (Figure 1b, Module 6). Nineteen ROIs, including 9 medial frontal regions, 1 temporal region, 7 parietal regions, 1 posterior cingulate cortex and 1 occipital region were clustered into one module forming the DMN (Figure 1b, Module 3). Because the frontal, striatal, and thalamic regions were separated into two modules, namely, the striatal-thalamic circuit and the DMN, we specified 2 sets of seed ROIs (11 seed ROIs for the striatal-thalamic circuit and 19 seed ROIs for the DMN) and performed one-sample *t* test separately. The 2 ROIs of the left middle temporal gyrus (Module 1) and the pons (Module 5) were removed in the subsequent statistical analysis because they were isolated from the modular structure. The details of the modular structure are listed in Table S2.

### 3.2 Significantly Existed ROI-based Functional Connectivity in Bipolar Patients and Healthy Controls

#### 3.2.1 Striatal-thalamic-related Functional Connectivity

**Figure 2:**
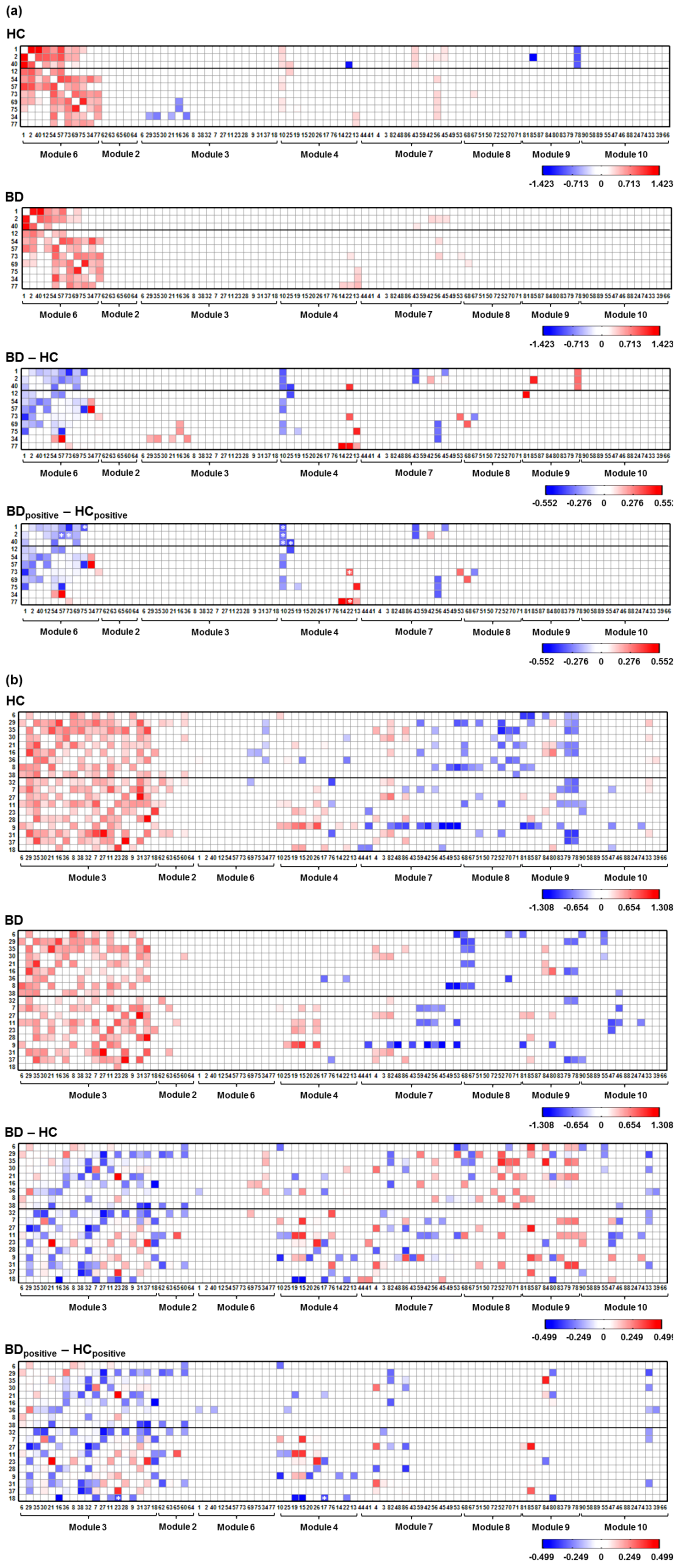
The significantly existed ROI-based functional connectivity in healthy subjects and bipolar patients and the correlation difference matrix between the bipolar and healthy groups. The x axis represents 88 ROIs (the 2 isolated ROIs, including the left middle temporal gyrus and the pons were removed), and the y axis represent the seed ROIs. (a) Striatal-thalamic-related Functional Connectivity. The first 3 seed ROIs are the striatal regions and the other are the thalamic regions. (b) DMN-related Functional Connectivity. HC_positive_ and BD_positive_ matrices were constructed by retaining the postive correlations. The significantly different connectivity in the BD_positive_ - HC_positive_ matrix was marked by a white asterisk (*).

The ROI-based functional connectivities related to the striatal-thalamic circuit (Module 6) exhibiting significant correlations which were significantly different from zero in each group are shown in Figure 2a. The first 3 seed ROIs (labeled 1, 2, and 40) in the y-axis of Figure 2a were the striatal regions, and the remaining 8 seed ROIs were the thalamic regions. Both groups presented significantly positive correlations within the striatal-thalamic circuit (Module 6). Several significantly positive correlations between the striatal regions and the anterior cingulate cortex (ROI 43 in Module 7) and between the striatal and thalamic regions and the posterior insula (ROI 56 in Module 7) were observed in both bipolar and healthy groups. The differences in the correlation results between the bipolar and healthy groups were displayed in the BD – HC difference matrix in Fig. 2a. It was worthy to note that the significantly positive correlations in individual groups would be conservative and the significantly negative correlations would be artifact due to our preprocessing strategy using global signal regression. Considering the limitations of global signal regression, we only retained the positive correlations to construct the HC_positive_ and BD_positive_ matrices. We have also displayed the differences between the HC_positive_ and BD_positive_ matrices (BD_positive_ - HC_positive_) to better illustrate the increase and decrease of correlations in bipolar patients without artifactual negative correlations (Fig. 2a). We then used the two-sample *t*-test as *P*<0.05 with an FDR correction for multiple comparisons to assess the differences of connectivities between the BD_positive_ and HC_positive_ matrices.

#### 3.2.2 DMN-related Functional Connectivity

The ROI-based functional connectivities related to the DMN (Module 3) exhibiting significant correlations in each group are shown in Figure 2b. The first 9 seed ROIs (labeled 6, 29, 35, 60, 21, 16, 36, 8, and 38) in the y-axis of Figure 2b were the medial frontal regions, and the remaining 10 seed ROIs included the temporal, parietal regions, posterior cingulate cortex and occipital region. The significantly positive correlations within the DMN were observed in both groups. Both groups exhibited a similar pattern of connectivity between the seed ROIs in DMN and ROIs in other modules. For example, several significantly positive connectivities were observed between seed ROIs in DMN and the retrosplenial and posterior cingulate cortices and the precuneus (ROI 19, 15, and 26 in Module 4) and between seed ROIs and the middle frontal gyrus (ROI 84 and 80 in Module 9). The correlation difference matrices (BD - HC and BD_positive_ - HC_positive_) related to the DMN was also shown in Fig. 2b.

### 3.3 Significant Changes in Striatal-thalamic-related Functional Connectivity for Bipolar Patients

The results obtained from the two-sample *t* test revealed that the bipolar patients presented significantly decreased and increased connectivities (marked by white asterisks in BD_positive_ - HC_positive_ matrix in Fig. 2a) related to the striatal-thalamic circuit, compared with the healthy controls. The significantly decreased functional connectivities were between the bilateral striatum regions (ROI 1, 2, and 40) and the middle as well as posterior cingulate cortex (ROI 10 and 25), and between the bilateral striatum (ROI 1, 2, and 40) and the mediodorsal as well as ventral posterolateral thalamus (ROI 57, 73, and 75). The part of anterior thalamus (ROI 1 and 2) also exhibited decreased connectivity with the middle cingulate cortex (ROI 10) as well as with the mediodorsal and ventral posterolateral thalamus (ROI 57, 73, and 75).

The significantly increased functional connectivities for the bipolar patients were between the left thalamus subregions (ROI 73 and 77) and the right parahippocampus (ROI 22). Table 2 shows the results of the significantly changed connectivity in the bipolar patients compared with healthy controls.

**Table 2:** Significantly changed connectivity related to the striatal-thalamic circuit in bipolar patients.

Significantly changed functional connectivity				Connectivity strength in healthy (mean+std)	Connectivity strength in bipolar (mean+std)	*p*-value (FDR corrected)
***Decrease in bipolar patients***						
1	L. vCAU, dPUT, aTHA 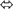	10	MCC	0.343±0.194	0.108±0.170	0.023
1	L. vCAU, dPUT, aTHA 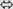	75	R. vpl THA	0.341±0.261	0.115±0.199	0.049
2	R. vCAU, dPUT, aTHA 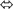	10	MCC	0.306±0.172	0.111±0.166	0.031
2	R. vCAU, dPUT, aTHA 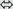	57	R. mdTHA	0.926±0.304	0.630±0.210	0.031
2	R. vCAU, dPUT, aTHA 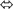	73	L. vpl THA	0.515±0.266	0.297±0.121	0.038
40	R. dCAU 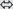	10	MCC	0.309±0.205	0.030±0.176	0.013
40	R. dCAU 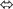	25	MCC, PCC	0.384±0.268	0.068±0.229	0.019
***Increase in bipolar patients***						
73	L. vpl THA 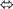	22	R. pvPHIP	0.044±0.220	0.248±0.162	0.042
77	L. puTHA 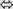	22	R. pvPHIP	0.133±0.227	0.338±0.166	0.038

### 3.4. Clinical Correlates of Striatal-thalamic-related Functional Connectivity

**Figure 3:**
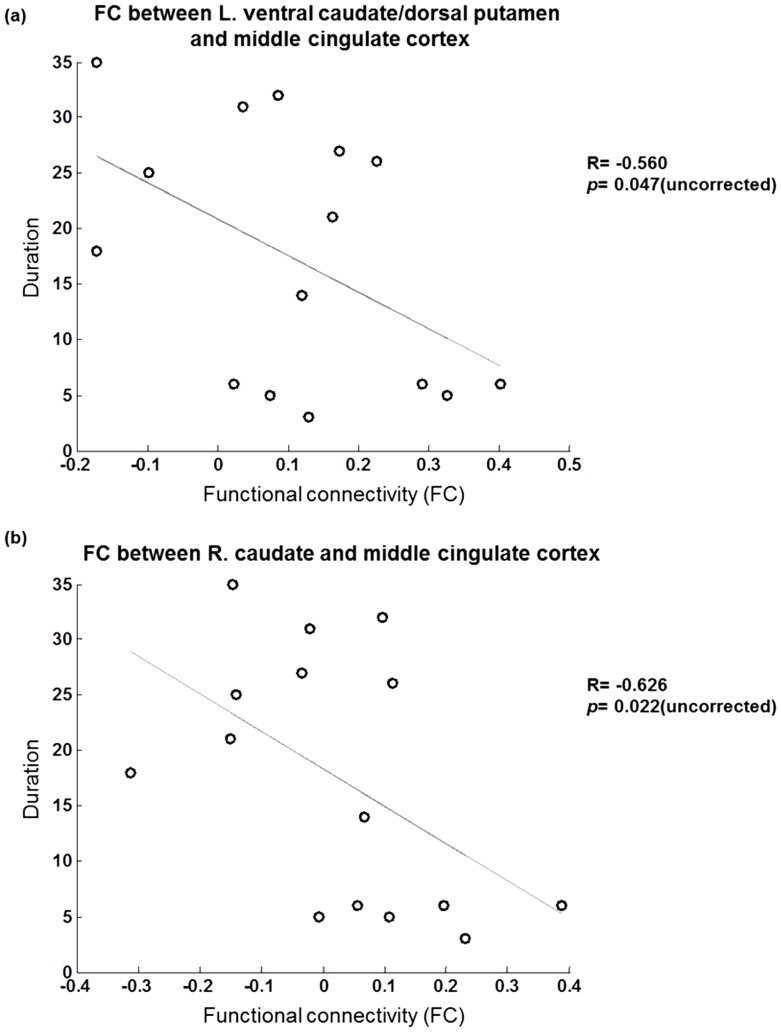
Clinical correlations between the altered functional connectivity and the clinical rating scales.

The relationships between the 9 significantly different connectivities between bipolar patients and healthy controls and 3 clinical measures were examined with correction for 9 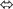 3 comparisons. The results of the clinical correlates indicated that the illness duration was negatively correlated with the functional connectivities between the striatal regions and the middle cingulate cortex, including that between the left striatal region and the middle cingulate cortex (R = −0.560, *P* = 0.047, uncorrected) and that between the right caudate and the middle cingulate cortex (R = −0.626, *P* = 0.022, uncorrected) (Figures 3a and 3b). Although the clinical correlations were insignificant after FDR corrections, the functional connectivities related to the striatal-thalamic circuit in the bipolar patients tended to decrease with the illness duration.

### 3.5. Significant Changes in DMN-related Functional Connectivity for Bipolar Patients

The results obtained from the two-sample *t* test revealed that no significant changes in frontal-related functional connectivity in the DMN module in the bipolar patients, compared with the healthy controls. Two significantly decreased functional connectivities (marked by white asterisks in BD_positive_ - HC_positive_ matrix in Fig. 2b) were observed between the left middle occipital gyrus (ROI 18 in Module 3) and the left posterior ventral parahippocampal gyurs (ROI 17 in Module 4) and between the left middle occipital gyrus and the right angular and middle occipital gyrus (ROI 23 in Module 3) in the bipolar patients. Based on the results of the one-sample *t* test, 201 DMN-related functional connectivities were significantly existed in bipolar and healthy groups. The *P* value of the two-sample *t* test after correction for 201 comparisons was considerably strict, that is the significant difference was identified if *P*<0.0002 = 0.05/201. Accordingly, only 2 significantly altered functional connectivity related to the DMN can be found in bipolar patients.

## Discussion

This study investigated the resting-state functional connectivity changes to reveal the abnormalities of a mental state without producing task biases for bipolar patients. We hypothesized that the resting-state functional connectivity related to the striatal-thalamic circuit and DMN might be altered in bipolar patients. Based on the results of the hierarchical modular analysis that the striatal and thalamic regions were clustered into one module in our resting data, we compared the connectivity related to the striatal-thalamic circuit between bipolar patients and healthy controls. The medial frontal regions were excluded from the module of the striatal and thalamic regions because the medial frontal regions exhibited strong connectivity with several parietal, temporal regions and the posterior cingulate cortex to form another module belonging to the DMN (Module 3 in Table S2). The strong correlations between the medial frontal regions and the striatal and thalamic regions might be observed when performing emotional or cognitive tasks rather than during the resting state [8], [9]. Finally, our results indicated that bipolar patients presented changed resting-state connectivity related to the striatal-thalamic circuit. The possible explanations for the altered resting-state connectivity of bipolar patients are discussed in the subsequent sections.

### 4.1 Connectivity Changes in the Resting State

Resting state data, which is measured by the absence of predetermined experimental task, provide several traits for probing psychiatric disorders. First, because bipolar disorder is related to internal anomalies in feeling and thinking, the resting state allowed bipolar patients to relax and spontaneously switch into self-referential representation reflecting their natural mental state without causing task-induced biases [18], [19]. Additionally, the resting-state connectivity can be altered when an individual suffers from a disease with a long duration [19]. Because the bipolar patients in this study suffered from several episodes of abnormal mood disturbances for many years (average duration: 17.3 years), the influence of long-term mood abnormalities might alter the resting-state functional circuits. Therefore, we used resting-state data to explore the brain functional alterations in bipolar patients.

### 4.2 Decreases in Striatal-related Functional Connectivity in Bipolar Patients

We observed that the bipolar patients exhibited significantly decreased striatal-related functional connectivity, including the connectivity between the striatum and thalamus as well as between the striatum and middle and posterior cingulate cortex (Table 2). The abnormalities of resting-state connectivity could be associated with the disrupted structural fiber integrity. The striatum has complex fiber connections with the thalamus, which relays subcortical input to the cortex (i.e., the cortico-striato-pallido-thalamic circuit) [41], [42]. Previous studies using diffusion weighted imaging have reported that bipolar patients exhibited a disrupted white matter structure (i.e., decreased fractional anisotropy to reflect the reduced fiber integrity) between the striatum and thalamus in the internal capsule [43], [44]. The disruption of fiber connections between the striatum and thalamus in bipolar patients can possibly cause decreased resting-state connectivity. Considering this basis of the structural deficits, the decreased resting-state functional connectivity related to the striatum and thalamus can be used as a probe to correlate with the task performance involving emotional and reward processes for bipolar patients [45].

Another significantly decreased functional connectivity in bipolar patients was observed between the striatum and the middle and posterior cingulate cortex (Table 2). A similar finding was reported in depressive patients by using resting-state fMRI [46]. Another study of healthy participants determined that the resting-state connectivity between the striatum and the middle and posterior cingulate cortex was associated with dopamine concentration [47]. Dopamine dysregulation has been hypothesized to be a factor in manic and depressive symptoms in bipolar patients [48], [49]. The dopamine dysregulation in the pathophysiology of bipolar disorder could be a reason for the reduced resting-state connectivity between the striatum and the middle and posterior cingulate cortex in the bipolar patients.

### 4.3 Increases in Thalamus-related Functional Connectivity in Bipolar Patients

Increased connectivity was observed between the pulvinar thalamus and the parahippocampus in the bipolar patients (Table 2). Both the pulvinar thalamus and parahippocampus have fiber connections with the amygdala [50]–[52]. The regions are involved in emotional processing, such as encoding and retrieving emotional memory [53]–[56]. Accordingly, 2 possible rationales for the increased connectivity between the pulvinar thalamus and the parahippocampus in the bipolar patients during resting state are as follows. First, participants were scanned at rest without external stimuli, and therefore, their attention was turned to the internal mental state involved in memory retrieval and self-perception. In addition, previous studies have reported that bipolar patients presented a tendency to easily engage with emotional memory, particularly negative-valence memory, rather than recalling memory with less emotional bias [57], [58]. Second, the bipolar patients exhibited frequent neural overactivation of the pulvinar thalamus and the parahippocampus during emotional processing [59]. This frequent coactivations between the regions might increase their associations [60], suggesting an enhanced synchronization among regions in the resting state.

### 4.4 Limitations and Methodological Concerns

Several limitations of this study must be mentioned. First, regarding the drug regimens for the bipolar group followed before their participation in this study, considering ethical concerns and clinical status of each patient, we required bipolar patients in a mood stable state to discontinue their medication for only 1 week before imaging. A 1-week interval from the final medication might not be sufficient to exclude the medication effect. However, the elimination half-life of the drugs for the bipolar group was below 30 hours. The plasma concentrations of the drugs were likely considerably low (<2%) after 7 days, which is sufficient to eliminate 97%–98% of drugs [61]. Therefore, the medication effects should have been minimal. Second, the correlations between the altered connectivity and clinical measures, including the illness duration and HAM-D scores, were significant only without correction for multiple comparisons, which might be attributable to the limited sample size. Further studies should include large sample sizes to assess the clinical correlates on resting-state functional connectivity in bipolar patients. Finally, neuropsychological testing was not performed in this study. The relationships between the altered resting-state functional connectivity and cognitive and behavioral deficits of bipolar patients must be further explored.

Three methodological concerns must also be considered. The first concern is related to the impact of head movement for each participant during scanning. Because a maximal translation of below 2 mm at each axis, and a rotation of below 2° were exhibited in each participant, the motion-related effects can be neglected. The second concern is the effect of global mean regression in the data preprocessing. Although global mean regression can introduce negative correlations, it can substantially suppress erroneously positive correlations [32]. Accordingly, we adopted the global mean regression in our data preprocessing and only retained the positive correlations to construct the connectivity matrices for both groups. The third concern is the a priori ROI selection. The conventional method of a priori ROI selection is subjective. To alleviate this problem, we adopted a resting-state fMRI template and hierarchical modular analysis so that the ROI selection can be more systematic.

## Conclusion

In summary, this study determined the altered resting-state connectivity related to the striatal-thalamic circuit for bipolar patients. The significantly different functional connectivity was in the striatal-thalamic circuit and between the circuit and other brain regions consisting of the middle and posterior cingulate cortex as well as the parahippocampus. Changes in the resting-state connectivity related to the striatal-thalamic circuit might be an inherent basis for the altered emotional and cognitive processing in bipolar patients.

## Supporting Information

Table S1 Anatomical locations, abbreviations and Brodmann areas of 90 functional ROIs.
(DOC)

Table S2 Ten modules resulted from the mean correlation matrix across all participants by using the hierarchical modular analysis.
(DOC)

## References

[1] Merikangas KR, Akiskal HS, Angst J, Greenberg PE, Hirschfeld RMA, et al (2007) LIfetime and 12-month prevalence of bipolar spectrum disorder in the national comorbidity survey replication. Archives of General Psychiatry 64: 543–552. 17485606 10.1001/archpsyc.64.5.543 PMC1931566

[2] Murray CJL, Lopez AD (1997) Global mortality, disability, and the contribution of risk factors: Global Burden of Disease Study. The Lancet 349: 1436–1442. 10.1016/S0140-6736(96)07495-8 9164317

[3] Bora E, Yucel M, Pantelis C (2009) Cognitive endophenotypes of bipolar disorder: A meta-analysis of neuropsychological deficits in euthymic patients and their first-degree relatives. Journal of Affective Disorders 113: 1–20. 18684514 10.1016/j.jad.2008.06.009

[4] Minassian A, Paulus MP, Perry W (2004) Increased sensitivity to error during decision-making in bipolar disorder patients with acute mania. Journal of Affective Disorders 82: 203–208. 15488248 10.1016/j.jad.2003.11.010

[5] Clark L, Iversen SD, Goodwin GM (2002) Sustained attention deficit in bipolar disorder. The British Journal of Psychiatry 180: 313–319. 11925353 10.1192/bjp.180.4.313

[6] Zubieta J-K, Huguelet P, O'Neil RL, Giordani BJ (2001) Cognitive function in euthymic Bipolar I Disorder. Psychiatry research 102: 9–20. 11368835 10.1016/s0165-1781(01)00242-6

[7] Metzger CD, van der Werf YD, Walter M (2013) Functional mapping of thalamic nuclei and their integration into cortico-striatal-thalamo-cortical loops via ultra-high resolution imaging—from animal anatomy to in vivo imaging in humans. Frontiers in neuroscience 7.. 10.3389/fnins.2013.00024 PMC3647142 23658535

[8] Haber SN, Calzavara R (2009) The cortico-basal ganglia integrative network: the role of the thalamus. Brain research bulletin 78: 69–74. 18950692 10.1016/j.brainresbull.2008.09.013 PMC4459637

[9] Price JL, Drevets WC (2012) Neural circuits underlying the pathophysiology of mood disorders. Trends in Cognitive Sciences 16: 61–71. 22197477 10.1016/j.tics.2011.12.011

[10] Haber SN, Kim K-S, Mailly P, Calzavara R (2006) Reward-related cortical inputs define a large striatal region in primates that interface with associative cortical connections, providing a substrate for incentive-based learning. The Journal of Neuroscience 26: 8368–8376. 16899732 10.1523/JNEUROSCI.0271-06.2006 PMC6673798

[11] Calzavara R, Mailly P, Haber SN (2007) Relationship between the corticostriatal terminals from areas 9 and 46, and those from area 8A, dorsal and rostral premotor cortex and area 24c: an anatomical substrate for cognition to action. European Journal of Neuroscience 26: 2005–2024. 17892479 10.1111/j.1460-9568.2007.05825.x PMC2121143

[12] Raichle ME, MacLeod AM, Snyder AZ, Powers WJ, Gusnard DA, et al (2001) A default mode of brain function. Proceedings of the National Academy of Sciences of the United States of America 98: 676–682. 11209064 10.1073/pnas.98.2.676 PMC14647

[13] Cerullo MA, Adler CM, Delbello MP, Strakowski SM (2009) The functional neuroanatomy of bipolar disorder. International Review of Psychiatry 21: 314–322. 20374146 10.1080/09540260902962107

[14] Li C-T, Hsieh J-C, Wang S-J, Yang B-H, Bai Y-M, et al (2012) Differential relations between fronto-limbic metabolism and executive function in patients with remitted bipolar I and bipolar II disorder. Bipolar Disorders 14: 831–842. 23167933 10.1111/bdi.12017

[15] Hwang J, In Kyoon L, Dager SR, Friedman SD, et al (2006) Basal Ganglia Shape Alterations in Bipolar Disorder. The American journal of psychiatry 163: 276–285. 16449482 10.1176/appi.ajp.163.2.276

[16] Lyoo IK, Sung YH, Dager SR, Friedman SD, Lee JY, et al (2006) Regional cerebral cortical thinning in bipolar disorder. Bipolar Disorders 8: 65–74. 16411982 10.1111/j.1399-5618.2006.00284.x

[17] Wilke M, Kowatch RA, DelBello MP, Mills NP, Holland SK (2004) Voxel-based morphometry in adolescents with bipolar disorder: first results. Psychiatry Research: Neuroimaging 131: 57–69. 15246455 10.1016/j.pscychresns.2004.01.004

[18] Chepenik LG, Raffo M, Hampson M, Lacadie C, Wang F, et al (2010) Functional connectivity between ventral prefrontal cortex and amygdala at low frequency in the resting state in bipolar disorder. Psychiatry Research: Neuroimaging 182: 207–210. 20493671 10.1016/j.pscychresns.2010.04.002 PMC2914819

[19] Öngür D, Lundy M, Greenhouse I, Shinn AK, Menon V, et al (2010) Default mode network abnormalities in bipolar disorder and schizophrenia. Psychiatry Research: Neuroimaging 183: 59–68. 20553873 10.1016/j.pscychresns.2010.04.008 PMC2902695

[20] Sheline YI, Price JL, Yan Z, Mintun MA (2010) Resting-state functional MRI in depression unmasks increased connectivity between networks via the dorsal nexus. Proceedings of the National Academy of Sciences 107: 11020–11025. 10.1073/pnas.1000446107 PMC2890754 20534464

[21] Anand A, Li Y, Wang Y, Lowe MJ, Dzemidzic M (2009) Resting state corticolimbic connectivity abnormalities in unmedicated bipolar disorder and unipolar depression. Psychiatry Research: Neuroimaging 171: 189–198. 19230623 10.1016/j.pscychresns.2008.03.012 PMC3001251

[22] Almeida JRC, Mechelli A, Hassel S, Versace A, Kupfer DJ, et al (2009) Abnormally increased effective connectivity between parahippocampal gyrus and ventromedial prefrontal regions during emotion labeling in bipolar disorder. Psychiatry Research: Neuroimaging 174: 195–201. 19910166 10.1016/j.pscychresns.2009.04.015 PMC2787954

[23] Wang F, Kalmar JH, He Y, Jackowski M, Chepenik LG, et al (2009) Functional and Structural Connectivity Between the Perigenual Anterior Cingulate and Amygdala in Bipolar Disorder. Biological psychiatry 66: 516–521. 19427632 10.1016/j.biopsych.2009.03.023 PMC2830492

[24] Versace A, Thompson WK, Zhou D, Almeida JRC, Hassel S, et al (2010) Abnormal Left and Right Amygdala-Orbitofrontal Cortical Functional Connectivity to Emotional Faces: State Versus Trait Vulnerability Markers of Depression in Bipolar Disorder. Biological psychiatry 67: 422–431. 20159144 10.1016/j.biopsych.2009.11.025 PMC2835157

[25] Shirer WR, Ryali S, Rykhlevskaia E, Menon V, Greicius MD (2012) Decoding Subject-Driven Cognitive States with Whole-Brain Connectivity Patterns. Cerebral Cortex 22: 158–165. 21616982 10.1093/cercor/bhr099 PMC3236795

[26] Meunier D, Lambiotte R, Fornito A, Ersche K, Bullmore ET (2009) Hierarchical modularity in human brain functional networks. Frontiers in Neuroinformatics 3.. 10.3389/neuro.11.037.2009 PMC2784301 19949480

[27] Blondel VD, Guillaume JL, Lambiotte R, Lefebvre E (2008) Fast unfolding of communities in large networks J Stat Mech. 2008: P10008.

[28] Hamilton M (1960) A RATING SCALE FOR DEPRESSION. J Neurol Neurosurg Psychiatry 23: 56–62. 14399272 10.1136/jnnp.23.1.56 PMC495331

[29] Young RC, Biggs JT, Ziegler VE, Meyer DA (1978) A rating scale for mania: reliability, validity and sensitivity. The British Journal of Psychiatry 133: 429–435. 728692 10.1192/bjp.133.5.429

[30] Vemuri P, Jones DT, Jack Jr CR (2012) Resting state functional MRI in Alzheimer's Disease. Alzheimer's research & therapy 4: 1–9. 10.1186/alzrt100 PMC3471422 22236691

[31] Liang X, Zou Q, He Y, Yang Y (2013) Coupling of functional connectivity and regional cerebral blood flow reveals a physiological basis for network hubs of the human brain. Proceedings of the National Academy of Sciences 110: 1929–1934. 10.1073/pnas.1214900110 PMC3562840 23319644

[32] Weissenbacher A, Kasess C, Gerstl F, Lanzenberger R, Moser E, et al (2009) Correlations and anticorrelations in resting-state functional connectivity MRI: A quantitative comparison of preprocessing strategies. NeuroImage 47: 1408–1416. 19442749 10.1016/j.neuroimage.2009.05.005

[33] Birn RM, Diamond JB, Smith MA, Bandettini PA (2006) Separating respiratory-variation-related fluctuations from neuronal-activity-related fluctuations in fMRI. NeuroImage 31: 1536–1548. 16632379 10.1016/j.neuroimage.2006.02.048

[34] Cordes D, Haughton VM, Arfanakis K, Carew JD, Turski PA, et al (2001) Frequencies Contributing to Functional Connectivity in the Cerebral Cortex in “Resting-state” Data. AJNR Am J Neuroradiol 22: 1326–1333. 11498421 PMC7975218

[35] Radhakrishna RC (1973) Linear statistical inference and its applications. New York: Wiley.

[36] Newman MEJ (2006) Modularity and community structure in networks. Proceedings of the National Academy of Sciences 103: 8577–8582. 10.1073/pnas.0601602103 PMC1482622 16723398

[37] Newman MEJ, Girvan M (2004) Finding and evaluating community structure in networks. Physical Review E 69: 026113. 10.1103/PhysRevE.69.026113 14995526

[38] Meunier D, Achard S, Morcom A, Bullmore E (2009) Age-related changes in modular organization of human brain functional networks. NeuroImage 44: 715–723. 19027073 10.1016/j.neuroimage.2008.09.062

[39] Benjamini Y, Hochberg Y (1995) Controlling the False Discovery Rate: A Practical and Powerful Approach to Multiple Testing. Journal of the Royal Statistical Society Series B (Methodological) 57: 289–300.

[40] Andrews-Hanna JR, Snyder AZ, Vincent JL, Lustig C, Head D, et al (2007) Disruption of Large-Scale Brain Systems in Advanced Aging. Neuron 56: 924–935. 18054866 10.1016/j.neuron.2007.10.038 PMC2709284

[41] Wakana S, Jiang H, Nagae-Poetscher LM, Van Zijl PC, Mori S (2004) Fiber Tract–based Atlas of Human White Matter Anatomy1. Radiology 230: 77–87. 14645885 10.1148/radiol.2301021640

[42] Alexander GE, DeLong MR, Strick PL (1986) Parallel organization of functionally segregated circuits linking basal ganglia and cortex. Annual Review of Neuroscience 9: 357–381. 10.1146/annurev.ne.09.030186.002041 3085570

[43] Sussmann JE, Lymer GKS, McKirdy J, Moorhead TWJ, Maniega SM, et al (2009) White matter abnormalities in bipolar disorder and schizophrenia detected using diffusion tensor magnetic resonance imaging. Bipolar Disorders 11: 11–18. 19133962 10.1111/j.1399-5618.2008.00646.x

[44] Haznedar MM, Roversi F, Pallanti S, Baldini-Rossi N, Schnur DB, et al (2005) Fronto-thalamo-striatal gray and white matter volumes and anisotropy of their connections in bipolar spectrum illnesses. Biological psychiatry 57: 733–742. 15820230 10.1016/j.biopsych.2005.01.002

[45] Phillips M, Ladouceur C, Drevets W (2008) A neural model of voluntary and automatic emotion regulation: implications for understanding the pathophysiology and neurodevelopment of bipolar disorder. Molecular psychiatry 13: 833–857. 10.1038/mp.2008.65 PMC2745893 18574483

[46] Bluhm R, Williamson P, Lanius R, Théberge J, Densmore M, et al (2009) Resting state default-mode network connectivity in early depression using a seed region-of-interest analysis: Decreased connectivity with caudate nucleus. Psychiatry and Clinical Neurosciences 63: 754–761. 20021629 10.1111/j.1440-1819.2009.02030.x

[47] Kelly C, de Zubicaray G, Di Martino A, Copland DA, Reiss PT, et al (2009) l-Dopa Modulates Functional Connectivity in Striatal Cognitive and Motor Networks: A Double-Blind Placebo-Controlled Study. The Journal of Neuroscience 29: 7364–7378. 19494158 10.1523/JNEUROSCI.0810-09.2009 PMC2928147

[48] Berk M, Dodd S, Kauer-Sant'Anna M, Malhi GS, Bourin M, et al (2007) Dopamine dysregulation syndrome: implications for a dopamine hypothesis of bipolar disorder. Acta Psychiatrica Scandinavica 116: 41–49. 10.1111/j.1600-0447.2007.01058.x 17688462

[49] Cousins DA, Butts K, Young AH (2009) The role of dopamine in bipolar disorder. Bipolar Disorders 11: 787–806. 19922550 10.1111/j.1399-5618.2009.00760.x

[50] Makris N, Meyer JW, Bates JF, Yeterian EH, Kennedy DN, et al (1999) MRI-Based Topographic Parcellation of Human Cerebral White Matter and Nuclei: II. Rationale and Applications with Systematics of Cerebral Connectivity. NeuroImage 9: 18–45. 9918726 10.1006/nimg.1998.0384

[51] Behrens TEJ, Johansen-Berg H, Woolrich MW, Smith SM, Wheeler-Kingshott CAM, et al (2003) Non-invasive mapping of connections between human thalamus and cortex using diffusion imaging. Nat Neurosci 6: 750–757. 12808459 10.1038/nn1075

[52] Aggleton J, Mishkin M (1984) Projections of the amygdala to the thalamus in the cynomolgus monkey. Journal of comparative neurology 222: 56–68. 6321564 10.1002/cne.902220106

[53] Pessoa L, Adolphs R (2010) Emotion processing and the amygdala: from a‘low road’to‘many roads’ of evaluating biological significance. Nature Reviews Neuroscience 11: 773–783. 20959860 10.1038/nrn2920 PMC3025529

[54] Phelps EA (2004) Human emotion and memory: interactions of the amygdala and hippocampal complex. Current Opinion in Neurobiology 14: 198–202. 15082325 10.1016/j.conb.2004.03.015

[55] Zihl J, Von Cramon D (1979) The contribution of the ‘second’visual system to directed visual attention in man. Brain 102: 835–856. 509197 10.1093/brain/102.4.835

[56] Burgess N, Maguire EA, O'Keefe J (2002) The human hippocampus and spatial and episodic memory. Neuron 35: 625–641. 12194864 10.1016/s0896-6273(02)00830-9

[57] Williams JMG, Barnhofer T, Crane C, Herman D, Raes F, et al (2007) Autobiographical memory specificity and emotional disorder. Psychological bulletin 133: 122. 17201573 10.1037/0033-2909.133.1.122 PMC2834574

[58] Clark DM, Teasdale JD (1982) Diurnal variation in clinical depression and accessibility of memories of positive and negative experiences. Journal of abnormal psychology 91: 87. 7200102 10.1037//0021-843x.91.2.87

[59] Delvecchio G, Fossati P, Boyer P, Brambilla P, Falkai P, et al (2012) Common and distinct neural correlates of emotional processing in bipolar disorder and major depressive disorder: a voxel-based meta-analysis of functional magnetic resonance imaging studies. European Neuropsychopharmacology 22: 100–113. 21820878 10.1016/j.euroneuro.2011.07.003

[60] Hebb DO (2002) The organization of behavior: a neuropsychological theory: L.Erlbaum Associates.

[61] Association AM (1993) Drug Evaluations: Annual 1993. Milwaukee: American Medical Association.

