# Supplementary material for: Altered Resting-State Functional Connectivity of Striatal-Thalamic Circuit in Bipolar Disorder
Source: PLoS One. 2014 May 2;9(5):e96422. doi: 10.1371/journal.pone.0096422 (PMC4008631; doi:10.1371/journal.pone.0096422)
Supplement: Table S1 — Anatomical locations, abbreviations and Brodmann areas of 90 functional ROIs. (DOC) [file pone.0096422.s001.doc]

**Table S1 Anatomical locations, abbreviations and Brodmann areas of 90 functional ROIs.**

| **ROI number** | **Regions in the functional networks** | **Abbreviations** | **Brodmann areas** |
| --- | --- | --- | --- |
| 1 | Left ventral Caudate, dorsal Putamen, anterior Thalamus | L.vCAU, dPUT, aTHA | N/A |
| 2 | Right ventral Caudate, dorsal Putamen, anterior Thalamus | R. vCAU, dPUT, aTHA | N/A |
| 3 | Left Inferior Frontal Gyrus | L. IFG | 45,48 |
| 4 | Right Inferior Frontal Gyrus | R. IFG | 45,48 |
| 5 | Pons | Pons | N/A |
| 6 | Medial Prefrontal Cortex, Anterior Cingulate Cortex, Orbitofrontal Cortex | MPFC, ACC, OFC | 9,10,24,32,11 |
| 7 | Left Angular Gyrus | L. ANG | 39 |
| 8 | Right Superior Frontal Gyrus | R. SFG | 9 |
| 9 | Posterior Cingulate Cortex, Precuneus | PCC, PCUN | 23,30 |
| 10 | Middle Cingulate Cortex | MCC | 23 |
| 11 | Right Angular Gyrus | R. ANG | 39 |
| 12 | Bilateral Anterior Thalamus | B. aTHA | N/A |
| 13 | Left ventral Hippocampus | L. vHIP | 20,36,30 |
| 14 | Right ventral Hippocampus | R. vHIP | 20,36,30 |
| 15 | Left Retrosplenial Cortex, Posterior Cingulate Cortex | L. RSC, PCC | 29,30,23 |
| 16 | Left Middle Frontal Gyrus | L. MFG | 8,6 |
| 17 | Left posterior ventral Parahippocampal Gyrus | L. pvPHIP | 37,20 |
| 18 | Left Middle Occipital Gyrus | L. MOG | 19,39 |
| 19 | Right Retrosplenial Cortex, Posterior Cingulate Cortex | R. RSC, PCC | 30,23 |
| 20 | Precuneus | PCUN | 7,5 |
| 21 | Right Superior Frontal Gyrus, Middle Frontal Gyrus | R. SFG, MFG | 9,8 |
| 22 | Right posterior ventral Parahippocampal Gyrus | R. pvPHIP | 37,30 |
| 23 | Right Angular Gyrus, Middle Occipital Gyrus | R. ANG, MOG | 39,19 |
| 24 | Right Lobule IX | R. Lobule IX | N/A |
| 25 | Middle Cingulate Cortex, Posterior Cingulate Cortex | MCC, PCC | 23 |
| 26 | Precuneus | PCUN | 7,19 |
| 27 | Left Angular Gyrus | L. ANG | 7,40 |
| 28 | Right Angular Gyrus | R. ANG | 7,40 |
| 29 | Left Middle Frontal Gyrus, Superior Frontal Gyrus | L. MFG, SFG | 8,9 |
| 30 | Left Inferior Frontal Gyrus, Orbitofrontal Gyrus | L. IFG, OFG | 45,47,10 |
| 31 | Left Superior Parietal Gyrus, Inferior Parietal Gyrus, Precuneus, Angular Gyrus | L. SPG, IPG, PCUN, ANG | 7,40,39 |
| 32 | Left Inferior Temporal Gyrus, Middle Temporal Gyrus | L. ITG, MTG | 20,37 |
| 33 | Right Crus I | R. Crus I | N/A |
| 34 | Left pulvinar Thalamus | L. puTHA | N/A |
| 35 | Right Middle Frontal Gyrus, Right Superior Frontal Gyrus | R. MFG, SFG | 46,8,9 |
| 36 | Right Middle Frontal Gyrus | R. MFG | 10,46 |
| 37 | Right Inferior Parietal Gyrus, Supramarginal Gyrus, Angular Gyrus | R. IPG, SMG, ANG | 7,40,39 |
| 38 | Right Superior Frontal Gyrus | R. SFG | 8 |
| 39 | Left Crus I, II, Lobule VI | L. Crus I, II, Lobule VI | N/A |
| 40 | Right dorsal Caudate | R. dCAU | N/A |
| 41 | Left Middle Frontal Gyrus | L. MFG | 9,46 |
| 42 | Left anterior Insula | L. aINS | 48,47 |
| 43 | Anterior Cingulate Cortex, Medial Prefrontal Cortex, Supplementary Motor Area | ACC, MPFC, SMA | 24,32,8,6 |
| 44 | Right Middle Frontal Gyus | R. MFG | 46,9 |
| 45 | Right anterior Insula | R. aINS | 48,47 |
| 46 | Left Lobule VI, Crus I | L. Lobule VI, Crus I | N/A |
| 47 | Right Lobule VI Crus I | R. Lobule VI, Crus I | N/A |
| 48 | Left Middle Frontal Gyrus | L. MFG | 46 |
| 49 | Left Supramarginal Gyrus, Inferior Parietal Gyrus | L. SMG, IPG | 40 |
| 50 | Left Precuneus | L. PCUN | 5 |
| 51 | Right Middle Cingulate Cortex | R. MCC | 23 |
| 52 | Right Superior Parietal Gyrus, Precuneus | R. SPG, PCUN | 7,5 |
| 53 | Right Supramarginal Gyrus, Inferior Parietal Gyrus | R. SMG, IPG | 2,40 |
| 54 | Left mediodorsal Thalamus | L. mdTHA | N/A |
| 55 | Lobule VI | Lobule VI | N/A |
| 56 | Left Posterior Insula, Putamen | L. pINS, PUT | 48 |
| 57 | Right mediodorsal Thalamus | R. mdTHA | N/A |
| 58 | Lobule VI | Lovule VI | N/A |
| 59 | Right Posterior Insula | R. pINS | 23 |
| 60 | Left Inferior Frontal Gyrus | L. IFG | 45,47 |
| 61 | Left Middle Temporal Gyrus | L. MTG | 21 |
| 62 | Left Middle Temporal Gyrus, Angular Gyrus | L. MTG, ANG | 21,37,39 |
| 63 | Left Middle Temporal Gyrus, Superior Temporal Gyrus, Supramarginal Gyrus, Angular Gyrus | L. MTG, STG, SMG, ANG | 21,22,42,40,39 |
| 64 | Right Inferior Frontal Gyrus (orbital part) | R. IFGorb | 47,45 |
| 65 | Right Supramarginal Gyrus, Superior Temporal Gyrus, Middle Temporal Gyrus | R. SMG, STG, MTG | 21,22,40 |
| 66 | Left Crus I | L. Crus I | N/A |
| 67 | Left Superior Temporal Gyrus, Heschl's Gyrus | L. STG, HES | 22,48 |
| 68 | Right Superior Temporal Gyrus | R. STG | 22,38,42,48 |
| 69 | Right ventral posterolateral Thalamus | R. vplTHA | N/A |
| 70 | Left Precentral Gyrus, Postcentral Gyrus | L. PreC, PostC | 4,3 |
| 71 | Right Precentral Gyrus, Postcentral Gyrus | R. PreC, PostC | 4,6,3 |
| 72 | Right Supplementary Motor Area | R. SMA | 6 |
| 73 | Left ventral posterolateral Thalamus | L. vpl THA | N/A |
| 74 | Bilateral Lobule IV, Lobule V, Lobule VI | B. Lobule IV, V, VI | N/A |
| 75 | Right ventral posterolateral Thalamus | R. vpl THA | N/A |
| 76 | Calcarine Sulcus | CAL | 17 |
| 77 | Left pulvinar Thalamus | L. puTHA | N/A |
| 78 | Left Middle Occipital Gyrus, Superior Occipital Gyrus | L. MOG, SOG | 18,19,17 |
| 79 | Right Middle Occipital Gyrus, Superior Occipital Gyrus | R. MOG, SOG | 17,18,19 |
| 80 | Left Middle Frontal Gyrus, Superior Frontal Gyrus, Precentral Gyrus | L. MFG, SFG, PreC | 6 |
| 81 | Left Inferior Parietal Sulcus | L. IPS | 2,40,7 |
| 82 | Left Inferior Frontal Gyrus (triangular and operculum part) | L. IFGtri, IFGoper | 44,48,45 |
| 83 | Left Inferior Temporal Gyrus | L. ITG | 37 |
| 84 | Right Middle Frontal Gyrus | R. MFG | 6 |
| 85 | Right Inferior Parietal Lobule | R. IPL | 2,40,7 |
| 86 | Right Inferior Frontal Gyrus (triangular and operculum part) | R. IFGtri, IFGoper | 44,48 |
| 87 | Right Middle Temporal Gyrus | R. MTG | 37 |
| 88 | Left Lobule VIII, Lobule VIIb | L. Lobule VIII, VIIb | N/A |
| 89 | Right Lobule VIII, Lobule VIIb | R. Lobule VIII, VIIb | N/A |
| 90 | Right Lobule VI, Crus I | R. Lobule VI, Crus I | N/A |
