# Supplementary material for: Altered Resting-State Functional Connectivity of Striatal-Thalamic Circuit in Bipolar Disorder
Source: PLoS One. 2014 May 2;9(5):e96422. doi: 10.1371/journal.pone.0096422 (PMC4008631; doi:10.1371/journal.pone.0096422)
Supplement: Table S2 — Ten modules resulted from the mean correlation matrix across all participants by using the hierarchical modular analysis. (DOC) [file pone.0096422.s002.doc]

**Table S2. Ten modules resulted from the mean correlation matrix across all participants by using the hierarchical modular analysis**.

| **Module number** | **ROI number** | **Regions** |
| --- | --- | --- |
| Module 1 |  |  |
|  | 61 | Left Middle Temporal Gyrus |
| Module 2 |  |  |
|  | 62 | Left Middle Temporal Gyrus, Angular Gyrus |
|  | 63 | Left Middle Temporal Gyrus, Superior Temporal Gyrus, Supramarginal Gyrus, Angular Gyrus |
|  | 65 | Right Supramarginal Gyrus, Superior Temporal Gyrus, Middle Temporal Gyrus |
|  | 60 | Left Inferior Frontal Gyrus |
|  | 64 | Right Inferior Frontal Gyrus (orbital part) |
| Module 3 |  |  |
|  | 6 | Medial Prefrontal Cortex, Anterior Cingulate Cortex, Orbitofrontal Cortex |
|  | 29 | Left Middle Frontal Gyrus, Superior Frontal Gyrus |
|  | 35 | Right Middle Frontal Gyrus, Superior Frontal Gyrus |
|  | 30 | Left Inferior Frontal Gyrus, Orbitofrontal Gyrus |
|  | 21 | Right Superior Frontal Gyrus, Middle Frontal Gyrus |
|  | 16 | Left Middle Frontal Gyrus |
|  | 36 | Right Middle Frontal Gyrus |
|  | 8 | Right Superior Frontal Gyrus |
|  | 38 | Right Superior Frontal Gyrus |
|  | 32 | Left Inferior Temporal Gyrus, Middle Temporal Gyrus |
|  | 7 | Left Angular Gyrus |
|  | 27 | Left Angular Gyrus |
|  | 11 | Right Angular Gyrus |
|  | 23 | Right Angular Gyrus, Middle Occipital Gyrus |
|  | 28 | Right Angular Gyrus |
|  | 9 | Posterior Cingulate Cortex, Precuneus |
|  | 31 | Left Superior Parietal Gyrus, Inferior Parietal Gyrus, Precuneus, Angular Gyrus |
|  | 37 | Right Inferior Parietal Gyrus, Supramarginal Gyrus, Angular Gyrus |
|  | 18 | Left Middle Occipical Gyrus |
| Module 4 |  |  |
|  | 10 | Middle Cingulate Cortex |
|  | 25 | Middle Cingulate Cortex, Posterior Cingulate Cortex |
|  | 19 | Right Retrosplenial Cortex, Posterior Cingulate Cortex |
|  | 15 | Left Retrosplenial Cortex, Posterior Cingulate Cortex |
|  | 20 | Precuneus |
|  | 26 | Precuneus |
|  | 17 | Left posterior ventral Parahippocampal Gyrus |
|  | 76 | Calcarine Sulcus |
|  | 14 | Right ventral Hippocampus |
|  | 22 | Right posterior ventral Parahippocampal Gyrus |
|  | 13 | Left ventral Hippocampus |
| Module 5 |  |  |
|  | 5 | Pons |
| Module 6 |  |  |
|  | 1 | Left ventral Caudate, dorsal Putamen, anterior Thalamus |
|  | 2 | Right ventral Caudate, dorsal Putamen, anterior Thalamus |
|  | 12 | Bilateral Anterior Thalamus |
|  | 40 | Right dorsal Caudate |
|  | 54 | Left mediodorsal Thalamus |
|  | 57 | Right mediodorsal Thalamus |
|  | 73 | Left ventral posterolateral Thalamus |
|  | 75 | Right ventral posterolateral Thalamus |
|  | 69 | Right ventral posterolateral Thalamus |
|  | 34 | Left pulvinar Thalamus |
|  | 77 | Left pulvinar Thalamus |
| Module 7 |  |  |
|  | 44 | Right Middle Frontal Gyus |
|  | 41 | Left Middle Frontal Gyrus |
|  | 4 | Right Inferior Frontal Gyrus |
|  | 3 | Left Inferior Frontal Gyrus |
|  | 82 | Left Inferior Frontal Gyrus (triangular and operculum part) |
|  | 48 | Left Middle Frontal Gyrus |
|  | 86 | Right Inferior Frontal Gyrus (triangular and operculum part) |
|  | 43 | Anterior Cingulate Cortex, Medial Prefrontal Cortex, Supplementary Motor Area |
|  | 59 | Right Posterior Insula |
|  | 42 | Left anterior Insula |
|  | 56 | Left Posterior Insula, Putamen |
|  | 45 | Right anterior Insula |
|  | 49 | Left Supramarginal Gyrus, Inferior Parietal Gyrus |
|  | 53 | Right Supramarginal Gyrus, Inferior Parietal Gyrus |
| Module 8 |  |  |
|  | 68 | Right Superior Temporal Gyrus |
|  | 67 | Left Superior Temporal Gyrus, Heschl's Gyrus |
|  | 51 | Right Middle Cingulate Cortex |
|  | 50 | Left Precuneus |
|  | 72 | Right Supplementary Motor Area |
|  | 52 | Right Superior Parietal Gyrus, Precuneus |
|  | 70 | Left Precentral Gyrus, Postcentral Gyrus |
|  | 71 | Right Precentral Gyrus, Postcentral Gyrus |
| Module 9 |  |  |
|  | 81 | Left Inferior Parietal Sulcus |
|  | 85 | Right Inferior Parietal Lobule |
|  | 87 | Right Middle Temporal Gyrus |
|  | 84 | Right Middle Frontal Gyrus |
|  | 80 | Left Middle Frontal Gyrus, Superior Frontal Gyrus, Precentral Gyrus |
|  | 83 | Left Inferior Temporal Gyrus |
|  | 79 | Right Middle Occipital Gyrus, Superior Occipital Gyrus |
|  | 78 | Left Middle Occipital Gyrus, Superior Occipital Gyrus |
| Module 10 |  |  |
|  | 90 | Right Lobule VI, Crus I |
|  | 58 | Lobule VI |
|  | 89 | Right Lobule VIII, Lobule VIIb |
|  | 55 | Lobule VI |
|  | 47 | Right Lobule VI Crus I |
|  | 46 | Left Lobule VI, Crus I |
|  | 88 | Left Lobule VIII, Lobule VIIb |
|  | 24 | Right Lobule IX |
|  | 74 | Bilateral Lobule IV, Lobule V, Lobule VI |
|  | 33 | Right Crus I |
|  | 39 | Left Crus I, II, Lobule VI |
|  | 66 | Left Crus I |
